# Incidence of advanced opportunistic infection and its predictors among HIV infected children at Debre Tabor referral Hospital and University of Gondar Compressive specialized hospitals, Northwest Ethiopia, 2020: A multicenter retrospective follow-up study

**DOI:** 10.1016/j.heliyon.2021.e06745

**Published:** 2021-04-09

**Authors:** Ermias Sisay Chanie, Wubet Alebachew Bayih, Binyam Minuye Birhan, Demeke Mesfin Belay, Getnet Asmare, Tegenaw Tiruneh, Yared Asmare Aynalem Aynalem, Biruk Beletew Abat, Sintayehu Asnakew, Maru Mekie, Getache Yideg Yitbarek, Fisha Alebel GebreEyesus

**Affiliations:** a Department of Pediatrics and Child Health Nursing, College of Health Sciences, Debre Tabor University, Debre Tabor, Ethiopia; b Department of Medical Laboratory, College of Health Sciences, Debre Tabor University, Debre Tabor, Ethiopia; c Department of Pediatrics and Child Health Nursing, College of Health Sciences, Debre Birhan University, Debre Birhan, Ethiopia; d Department of Nursing, College of Health Sciences, Woldia University, Woldia, Ethiopia; e Department Psychiatric, School of Medicine, College of Health Sciences, Debre Tabor University, Debre Tabor, Ethiopia; f Department Midwifery, College of Health Sciences, Debre Tabor University, Debre Tabor, Ethiopia; g Department of Biomedical, College of Health Sciences, Debre Tabor University, Debre Tabor, Ethiopia; h Department Nursing, College of Health Sciences, Wolkite University, Wolkite, Ethiopia

**Keywords:** Advanced, Ethiopia, Opportunistic infection, Predictors, Time to develop

## Abstract

**Background:**

This study is aimed to assess the incidence of advanced opportunistic infections (OIs) and its predictors among Human Immunodeficiency Virus (HIV) infected children at Debre Tabor referral Hospital and University of Gondar Compressive specialized Hospitals, Northwest Ethiopia, 2020.

**Methods:**

A retrospective follow-up study was conducted from June 1, 2010, to May 30, 2020. A total of 349 children under the age of 15 who had received Anti-Retroviral Therapy (ART) were included in the study. Data were entered into Epi info version 7.2 and then exported to Stata 14.0 for analysis. Kaplan Meier curve and Log-rank test were used to determine the median survival time and the discrepancy of different categorical variables. The Cox regression model was used to identify the predictors of advanced opportunistic infections. The Adjusted hazard ratio (AHR) at 95% confidence interval (CI) was used to declare statistical significance.

**Result:**

The incidence rate of advanced opportunistic infection was 5.53 per 100 (95% CI: 4.7, 6.9) Person per year observation (PYO). The median survival time was 113 months and the total follow-up periods were yielding 18882 months. Children presenting with treatment failure, Cotrimoxazole Preventive Therapy (CPT) non-user, low hemoglobin level (<10 mg/dl), and poor/fair level of adherence to ART were less free survival time as compared to their counterparts for advanced opportunistic infections.

**Conclusion:**

In this study, the median of advanced OIs free survival time was found to be low and the incidence rate was found to be high. The incidence advanced OIs was associated with anemia, treatment failure, and poor/fair level of adherence, cotrimoxazole preventive therapy non-users. Further research should conduct to evaluate and to improve the quality of care in the study area.

## 1 Introduction

Opportunistic infections (OIs) are infections that occur more often or are more severe in people with weakened immune systems [1], including people with Human Immunodeficiency Virus (HIV) [2]. In 2019, 36.2 million were adults and 1.8 million were children (<15 years old) living with HIV. The same year, there 690 000 were Acquired Immune Deficiency Syndrome (AIDS) related deaths, of which the majority can be attributed to OIs [3, 4]. Besides, advanced OIs is the major cause of disease and death in HIV infected children [5].

The hallmark of HIV infection is immunosuppression which predisposes to OIs, which contributes to morbidity and mortality in HIV infected children worldwide [5, 6, 7]. As a result, the prevention of opportunistic infections in HIV infected children is crucial [8, 9, 10]. However, OIs remain a major cause of morbidity and mortality among these vulnerable population even after Anti-Retroviral Therapy (ART) [7].

In resource-rich countries, the life expectancy of newly infected patients with HIV starting on ART has almost reached the lifespan of the general population [11, 12, 13]. However, in Sub-Saharan countries, life expectancy among HIV infected patients is far from the general population [7, 12, 14], due to OIs [15]. Regardless of increasing access to effective HIV prevention, diagnosis, and treatment worldwide [16], OIs are the leading causes of morbidity and mortality among HIV-infected children [17, 18].

The impact of HIV associated OIs in HIV-infected children has been well documented in advanced countries [19], while in Sub -Saharan Africa (SSA), the true burden of OIs among HIV-children remains poorly documented [20], and Ethiopian is one of them [5].

Despite the incidence of OIs after initiation of ART is a decline dramatically worldwide [13, 19], they are a leading cause of poor quality of life, hospitalization, and poor adherence among HIV infected children in Ethiopia [5, 21, 22]. Besides, OIs remains a major cause of morbidity and mortality among this vulnerable population [7], which contributes to 94.1% of HIV-related deaths [17, 18]. Advanced OIs are a life-threatening cause of morbidity and mortality associated with HIV [6], markedly in HIV infected children [23, 24]. since their immunity is not well developed [25].

Even advanced OIs is a common cause of hospitalization and death among HIV infected children, there has been no prior evidence about the time to develop advanced opportunistic infections and its predictors among HIV infected children in Ethiopia. Therefore, this study is aimed to assess the incidence of advanced opportunistic infection and its predictors among HIV infected children at Debre Tabor referral Hospital and University of Gondar Compressive Specialized Hospital, Northwest Ethiopia, 2020.

## 2 Materials and methods

### 2.1 Study design and period

A retrospective follow-up study was conducted from June 1, 2010, to May 30, 2020, at University of Gondar Compressive specialized hospital and Debre Tabor referral hospital, Northwest Ethiopia, 2020.

### 2.2 Study setting

The first study area was University of Gondar Compressive specialized hospital pediatric ART clinic which is Gondar town, which is located in North West Ethiopia. It is far from 737 km from Addis Ababa, Ethiopia. The hospital serves about 5 million people. ART service has been started in 2005. In 2017, a total of 8581 adults and 1138 children were registered in ART clinic [26]. Besides, the second study area was Debre Tabor referral hospital which is located in Debre Tabor town and far from 665 km from Addis Ababa. The hospital serves around 3.7 million people. ART service is one of the services delivered since 2005. In 2018, a total of 9859 adults and 698 pediatrics patients have been enrolled in ART clinic [27].

### 2.3 Source population

All HIV infected children at Debre Tabor referral hospital and university of Gondar compressive specialized hospital, 2020.

### 2.4 Study population

All HIV infected children from June 1, 2010, to May 30, 2020, at Debre Tabor referral hospital and University of Gondar compressive specialized hospital, 2020.

### 2.5 Inclusion criteria

Children whose age less than 15 year and followed their treatment during the study period were eligible for this study.

### 2.6 Exclusion criteria

Unrecorded of the outcome variable (i.e., advanced OI was not recorded) in the medical charts and ART follow-up database of HIV infected children were excluded.

### 2.7 Operational definition

**Time to develop advanced opportunistic infection**: The time from children ART initiation to the occurrence of the event (i.e., advanced OIs) during the follow-up period.

**Advance opportunistic infection (event):** They are a severe type of opportunistic infections associated with HIV [5, 23, 24]. According to the Ethiopian ART guidelines, advanced OIs include bacterial pneumonia, pulmonary TB, extra-pulmonary TB, oral candidiasis, esophageal candidiasis, and chronic diarrhea for longer than 1 month, pneumocystis pneumonia, toxoplasmosis, Cryptococci meningitis, non-Hodgkin's lymphoma, Kaposi's sarcoma, wasting syndrome and others [28, 29].

**HIV infected children** reference the age of the children <15 years, and they enrolled in pediatric ART clinic in Ethiopian context [30].

**Censored:** Children who were lost to follow-up, transferred out to another health institution, and end of the study period before developing an advanced opportunistic infection.

**Level of adherence to ART** was classified into good, fair, or poor by the percentage of drug dosage calculated from the total monthly doses of ART drugs taking (Good >95%, fair 85–94%, poor <85%) [29].

**Underweight or stunting** was defined as weight for age Z-score < −2 SD for under-five children and BMI for age Z-score < −2 SD for older children [17].

**Low hemoglobin level (Anemia)** was defined as having a hemoglobin level <10 mg/dl [17].

### 2.8 Variables

**Dependent variable:** Time to develop advanced OIs.

**Independent variables:**

**Socio-demographic characteristics**: Age, sex, residence, HIV disclosure status, and religion. Moreover, marital status, educational status, occupation, life status, and HIV status of the caregiver.

**Clinical and Treatment-related characteristics:** Weight for height, height for age, low hemoglobin level, CD4 (Cluster of Differentiation 4) counts or %, regimen at baseline, Cotrimoxazole preventive therapy (CPT), Isoniazid preventive therapy (IPT), level of adherence to ART, treatment failure, functional status, year of initiation and duration on ART.

### 2.9 Sample size determination

Log-rank survival data analysis of the two-population proportion formula was used to determine the sample size. Besides, the assumptions- 95% Confidence Interval (CI), 80% optimum statistical power, and taking type one error 5% were taken with hemoglobin level < 10 g/dl as the exposed group denoted by q1 (0.548) and hemoglobin level ≥10 g/dl as the non-exposed denoted by q0 (0.7020) from a study that was conducted at Debre Markos referral hospital [17], and the final total sample size, after adding 10% as incomplete or inconsistent data were 349.

### 2.10 Sampling procedure

**Figure 1:**
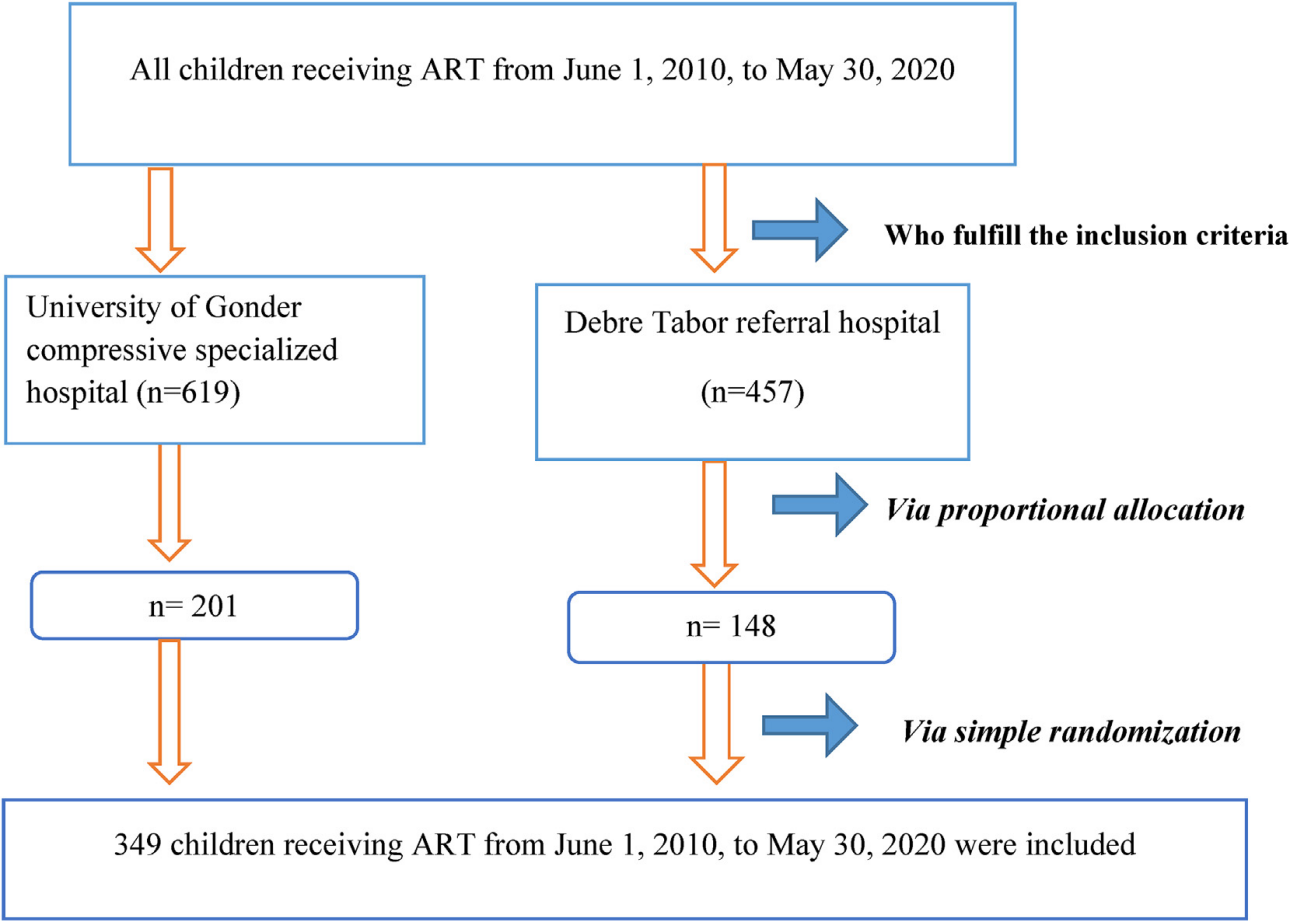
Schematic diagram of sampling procedure predictors among HIV infected children at Debre Tabor referral hospital and university of Gondar compressive specialized hospital, 2020 (n = 349).

A total of 1076 children from June 1, 2010, to May 30, 2020 were selected from both hospitals since they fulfilled the inclusion criteria. By using proportional allocation, 148 and 201children were selected followed by a simple random sampling technique from Debre Tabor referral hospital and University of Gondar compressive specialized hospital, respectively (Figure 1).

### 2.11 Data collection tools and procedures

The data was collected from the ART follow-up database and children's chart and data extraction tool was adapted from Ethiopian ART guidelines. The socio-demographic, clinical, and treatment-related variables were extracted from the ART follow-up database and medical records by five bachelor degree nurses who were familiar with the ART follow-up. The two supervisors had overseen the overall process of data collection, and the data collector and supervisors were taking basic ART training.

### 2.12 Data quality control

The data extraction tool was pretested through 5% (18 medical records of the sample to check the consistency and completeness of the data items). Moreover, the data extraction tool was adopted from a standardized Ethiopian ART guidelines follow-up format. Two days of training were given on how to review ART follow-up databases and medical records plus the objective of the study for data collectors and supervisors. Besides, the data collector coded the charts to avoid duplication, and charts that didn't fulfill the inclusion criteria were excluded.

### 2.13 Data processing and analysis

Before the actual entry, data were checked for inconsistency, coding errors, completeness, accuracy, clarity, and missing values; and then entered into Epi Info 7.2 and analyzed by Stata14.0 Software. Descriptive and summary statistics were carried out using the median, mean, proportion, frequency, and interquartile ranges. Tables and figures were used to present the data. The incidence of advanced opportunistic infection was calculated by dividing the number of children developing Advanced OIs during the follow-up period by the children person-years observation. A Kaplan Meier curve was used to estimate the overall median survival time. In addition to this, the Kaplan Meier curve was used to determine the median survival time with respect to different categories. A Log-rank test was used to see the discrepancy between the predictor variables along with the p-value. COX proportional hazard model assumption and goodness of fitness were checked through the Schoenfeld residual (global test = 0.95800) and stphplot-Log-Log parallel plot of survival respectively. Moreover, the Cox proportional hazard model was fitted both bivariable and multivariable to identify predictors of advanced OIs. The variables having a p-value up to 0.25 in the bivariable analysis were entered into the multivariable model to declare a significant variable. Adjusted Hazard ratio (AHR) with 95% confidence interval was used to explain the strength of the association, and variables with P-value less than 0.05 in the multivariable analysis were considered as significantly associated with the dependent variable.

**Ethical considerations:** Ethical approval was obtained from Debre Tabor University ethical review committee. Besides, permission letters from Debre Tabor referral hospital and University of Gondar compressive specialized hospital were obtained from ART focal person. Name or identification numbers of children were not allowed to record in the data extraction tool, rather grouping analysis was carried out to ensure the confidentiality of the study participants.

## 3 Results

### 3.1 Socio-demographic characteristics of the children

Of the 349 participants, 180 (51.6%) were male with a mean ± SD age of 7.33 ± 0.178 years; 188 (53.9%) of the participants were between 5- 9 years of age. Fifty-one percent of children knew their HIV status, and 68.5% of their caregivers were alive. 54.2% and 77.7% of child caregivers were none employed and HIV positive respectively. Moreover, the majority of the caregivers (79.91%) were rural residences, 67.0% were married, and 85.1% were orthodox Christian in the follow-up period (Table 1).

**Table 1:** Socio demographic characteristics of the study participants.

Variables		Frequency	Percent
Age	0–4	105	30.1
	5–9	188	53.9
	10–14	56	16.0
Sex	Male	180	51.6
	Female	169	48.4
Residence	Rural	70	20.1
	Urban	279	79.9
Marital status of the caregiver	Single	10	3.0
	Married	234	67.0
	Widowed/Divorced	105	30.0
Religion	Orthodox	297	85.1
	Muslim	47	13.5
	Other∗	5	1.4
Caregiver's educational status	Can't read and write	143	41.0
	primary school (1–8)	113	32.4
	Secondary school (9–12)	93	26.6
Occupation of the caregiver	Un employed	228	54.2
	Merchant	33	9.5
	Governmental employed	77	22.1
	Non-governmental Employed	11	3.2
Parental status of child	Both alive	239	68.5
	One or both deceased	110	31.5
HIV status of the parents/caregiver	Positive	271	77.7
	Negative	38	10.9
	Unknown	40	11.5
HIV disclosure Status	Yes	178	51.0
	No	171	49.0

Others∗ = protestant and catholic.

### 3.2 Clinical and treatment-related characteristics

The majority (84%) of the children were hemoglobin level ≥ 10 mg/dl and two-thirds (63.0) of the children were initiated ART after the year 2014. About 80.2% of children had CD4 counts or % above the threshold level, 86.5% of children were Zidovudine containing regimen. Almost half (51. %) of the children were underweight and 41.0% of the children were stunted. Besides, 78.5% and 28.7 % of children were CPT and IPT users respectively. The majority, 91.7% and 76.8 % of children were appropriate functional status and a good level of ART adherence respectively. 41. % of children were taking ART more than 59 months, whereas 12.6 % had treatment failure (Table 2).

**Table 2:** Clinical and treatment-related characteristics of the children.

Variables		Frequency (n = 349)	Percent (%)
Weight for height	Normal	171	49.0
	Underweight	178	51.0
Height for age	Normal	206	59.0
	Stunted	143	41.0
Hemoglobin level	<10 mg/dl	56	16.0
	≥10 mg/dl	293	84.0
CD4 counts or %	Below threshold	69	19.8
	Above threshold	280	80.2
Regimen at baseline	Zidovudine contain	302	86.5
	Non-zidovudine contains	47	13.5
Cotrimoxazole preventive therapy (CPT)	Yes	274	78.5
	No	75	21.5
Isoniazid preventive therapy (IPT)	Yes	100	28.7
	No	249	71.3
Level of adherence to ART	Good	268	76.8
	Poor/fair	81	23.2
Treatment failure	Yes	44	12.6
	No	305	87.4
Functional status	Appropriate	320	91.7
	Delayed/Regressing	29	8.3
Year initiation	<2014	220	63.0
	≥2014	129	37
Duration on ART	<36 months	112	32.1
	36–59 months	94	26.9
	>59 months	143	41.0
Follow-up status of advanced OI	Yes	87	24.9
	No	262	75.1

### 3.3 Incidence of advanced opportunistic infections during follow-up

**Figure 2:**
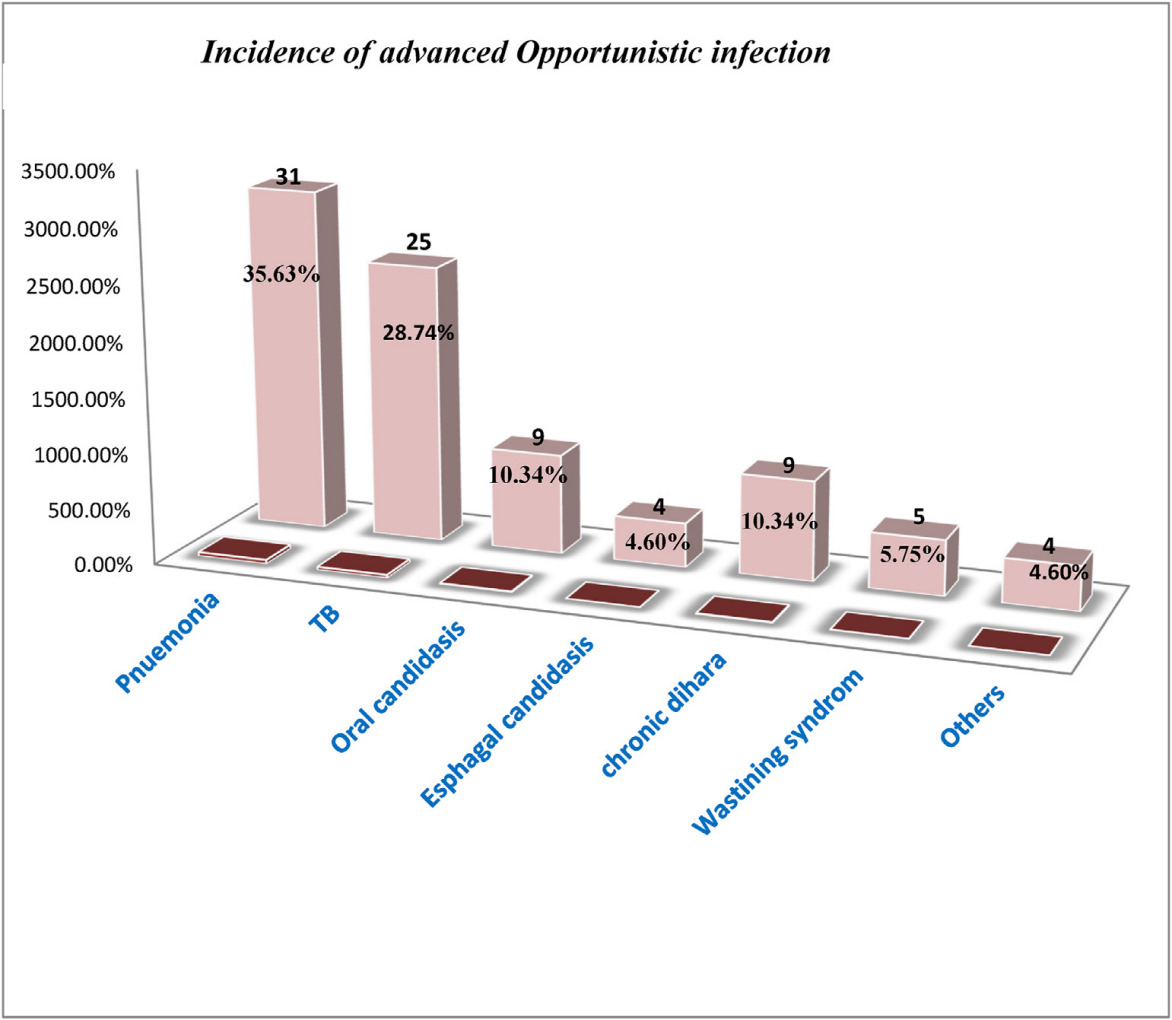
Incidence advanced Opportunistic infection among HIV infected children at Debre Tabor referral hospital and university of Gondar compressive specialized hospital, 2020 (n = 349).

The cumulative probability of survival of advanced OIs was found to be 24.9% [CI: 23.2–29.5]. Besides, the incidence rate of advanced OIs was found to be 5.53 per 100 PYO (95% CI: 4.7, 6.9). Pneumonia was the most common complaint seen in 35.6% of the children followed by tuberculosis (28.7%), oral candidiasis (10.3 %), esophageal candidiasis (4.6%), wasting syndrome (5.8%), chronic diarrhea greater than 01 months (10.3%), and others (16%) (Figure 2).

### 3.4 Kaplan-Meier advanced opportunistic infections free survival time

**Figure 3:**
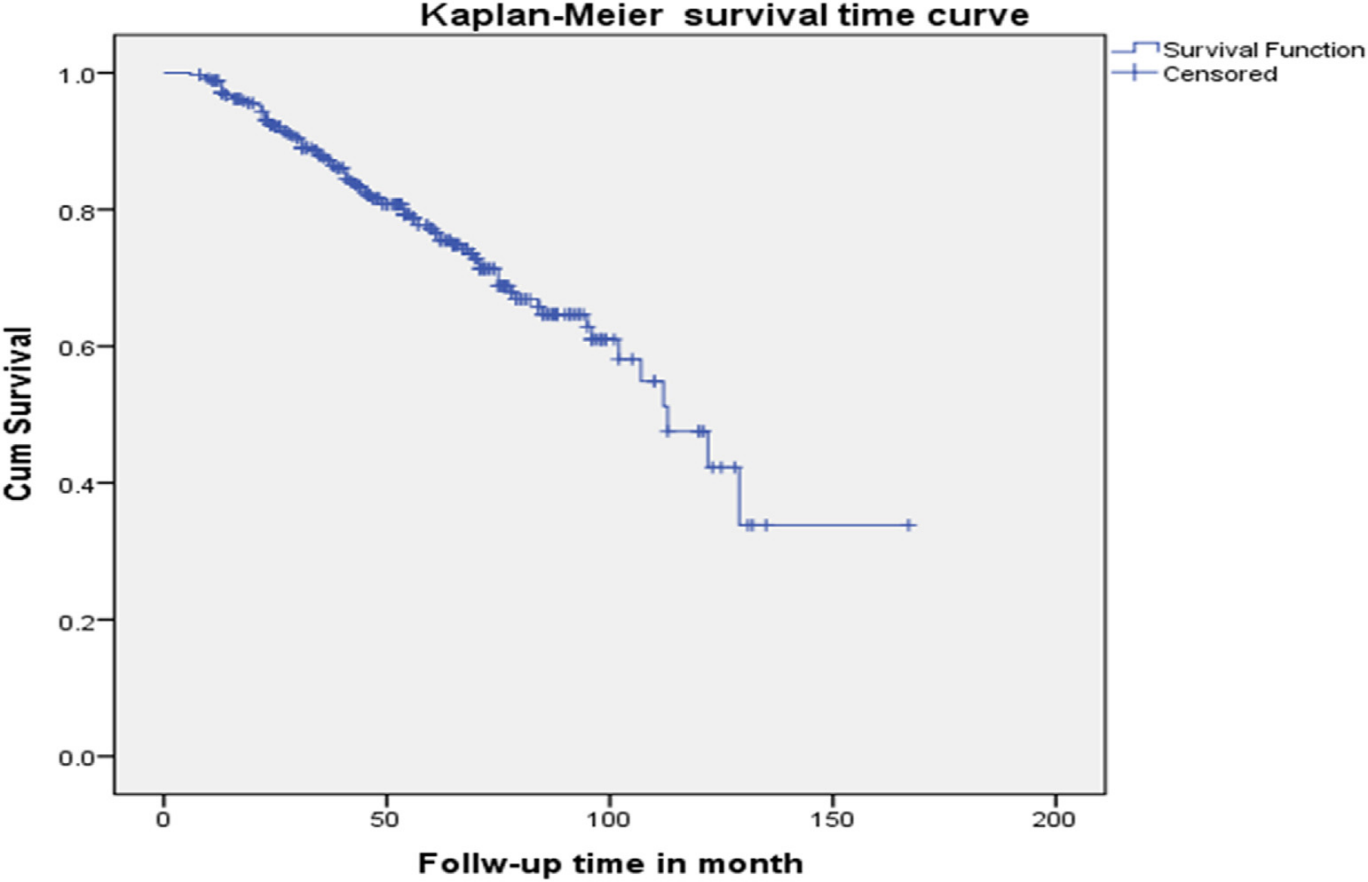
Kaplan-Meier of advanced Opportunistic infection -free survival time among HIV infected children at Debre Tabor referral hospital and university of Gondar compressive specialized hospital, 2020 (n = 349).

In this study, the median advanced OIs free survival time was 113 months (IQR = 65,161). In addition to this, the total follow-up period yields were 18882 months or 1573.5 years (figure). The advanced OIs free survival probability of children at the end was 0.75 [95% CI: 0.72–0.70] follow-up period (Figure 3).

### 3.5 Advanced opportunistic infections free survival time of predictor variables

**Figure 4:**
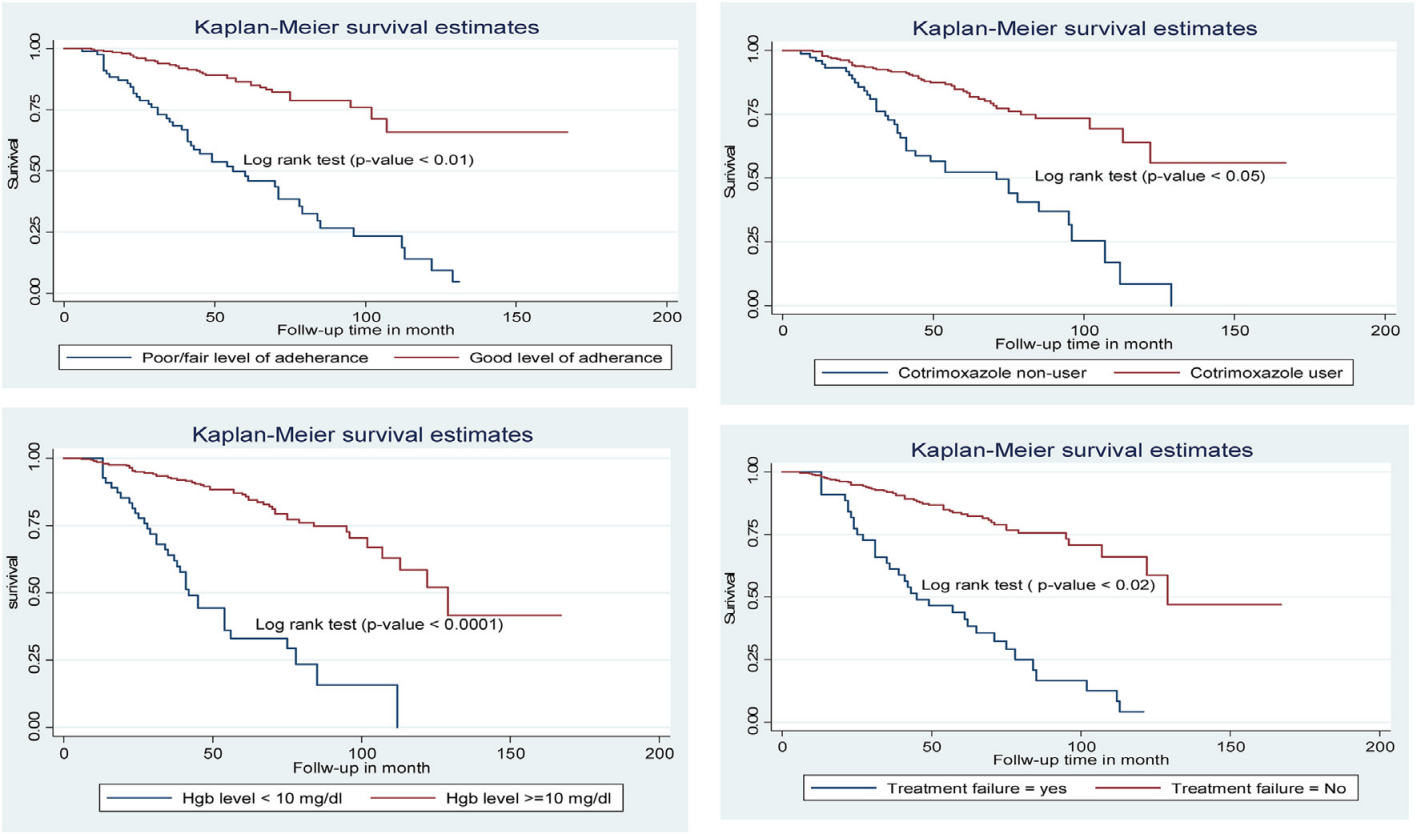
Kaplan-Meier of advanced Opportunistic infection -free survival time by level of adherence to ART and Hemoglobin level among HIV infected children at Debre Tabor referral hospital and university of Gondar compressive specialized hospital, 2020 (n = 349). Kaplan-Meier of advanced Opportunistic infection -free survival time by Cotrimoxazole preventive therapy, and treatment failure predictor variable among HIV infected children at Debre Tabor referral hospital and university of Gondar compressive specialized hospital, 2020 (n = 349).

The advanced OIs free survival time of children after treatment failure were less free survival time 45 [95% CI: 31–71] months as compared with their counterparts 129 months [95% CI: 122–134]. Additionally, children who were CPT non-user were less free survival time for advanced OIs 71 [95% CI: 41–95] months as compared with their counterparts 113 [95% CI: 106–117] months (Figure 4). Besides, children presenting with poor/fair adherences were less free survival time for advanced OIs 56 [95% CI: 41–78] months as compared with children with good level adherence 115 [95% CI: 107–123] months and children with low hemoglobin level (<10 mg/dl) were less free survival time for advanced OIs 42 [95% CI: 35–54] months as compared with their counterparts 129 [95% CI: 107–142] months (Figure 4).

### 3.6 Predictors of advanced opportunistic infections among HIV-infected children on ART

Residence, marital status of the caregivers, hemoglobin level, weight for age, taking CPT prophylaxis, treatment failure, and level of adherence to ART adherence were variables entered into the multivariable analysis. Of these, treatment failure, CPT non-users, low hemoglobin level, and poor/fair level of adherence to ART were found to be significant predictors for advanced OIs occurrence. Children who were low hemoglobin levels (<10 mg/dl) were nearly 3 times [AHR: 2.8, 95% CI: 1.70, 4.64] more likely to develop advanced OIs as compared to those children with hemoglobin levels (≥10 mg/dl). Besides, children who were CPT non-user were nearly 2 times [AHR: 1.7, 95% CI: 1.1, 2.8] more likely to develop advanced OIs as compared to those children with CPT users. Children presenting with treatment failure were nearly 2 times [AHR: 1.9, 95% CI: 1.1, 3.3] a higher risk of developing advanced OIs as compared to the counterpart. Likewise, children who were poor/fair level of adherence were nearly 2 times [AHR: 1.6, 95% CI: 1.1, 3.3] a higher risk of developing advanced OIs as compared to the counterpart (Table 3).

**Table 3:** Bi-variable and multivariable Cox-regression analysis of predictors for advanced opportunistic infections.

Variables		Advance OI		OR (95% CI)		
		Yes (n = 87)	No (=262)	CHR	AHR	P-value
Age of the child (years)	0–4	23	82	1.2 (0.61–2.40)		
	5–9	51	137	1.3 (0.71–2.42)		
	10–14	13	43	1		
Sex	Male	46	134	1.1 (0.73–1.70)		
	Female	41	128	1		
Residence	Rural	21	49	1.3 (0.82–2.20)	1.2 (0.72–2.14)	0.431
	Urban	66	213	1		
Marital status of the caregiver	Single	8	12	2.6 (1.20–5.82)	1.4 (0.60–3.22)	0.475
	Married	54	171	1.1 (0.71–1.84)	1.1 (0.68–1.87)	0.636
	Widowed/Divorced	25	78	1		
Religion	Orthodox	71	226	1		
	Muslim	14	33	1.5 (0.84–2.67)		
	Other∗	2	3	1.4 (0.35–5.95)		
Caregiver's educational status	Can't read and write	33	110	1.2 (0.67–2.01)		
	primary school (1–8)	32	81	1.3 (0.78–2.32)		
	Secondary school (9–12)	22	71	1		
Occupation of the caregiver	Housewife	166	62	0.7 (0.22–2.22)		
	Merchant	28	5	0.5 (0.11–1.90)		
	Governmental employ	60	17	0.5 (0.20–1.77)		
	NON-governmental Employ	8	3	1		
Parental status of child	Both alive	58	181	1.1 (0.67–1.65)		
	One or both deceased	29	81	1		
HIV status of the caregiver	Positive	66	205	0.9 (0.47–1.76)		
	Negative	11	27	1.2 (0.50–2.76)		
	Unknown	10	30	1		
HIV disclosure Status	Yes	46	125	1		
	No	41	137	0.9 (0.61–1.43)		
Variables		Advance OI		OR (95% CI)		
		Yes (n = 87)	No (=262)	COR	AOR	P-value

Weight for height	Normal	33	138	1		
	Underweight	54	124	1.7 (1.13–2.70)	1.3 (0.84–2.15)	0.215
Height for age	Normal	49	157	1		
	Stunted	38	105	1.1 (0.74–1.73)		
Hemoglobin level	<10 mg/dl	36	20	5.9 (3.80–9.11)	2.8 (1.70–4.64)	0.000∗∗
	≥10 mg/dl	51	242	1	1	
CD4 counts or %	Below threshold	19	50	1.2 (0.72–1.98)		
	Above threshold	68	212	1		
Regimen at baseline	Zidovudine contains	75	227	0.9 (0.49–1.67)		
	Non-zidovudine contains	12	35	1		
Cotrimoxazole preventive therapy (CPT)	Yes	48	226	1	1	
	No	39	36	3.7 (2.42–5.65)	1.7 (1.1–2.80)	0.040 ∗
Isoniazid preventive therapy (IPT)	Yes	187	62	1		
	No	75	25	1.0 (0.60–1.53)		
ART adherence	Good	39	229	1	1	
	Poor/fair	48	33	5.0 (3.24–7.60)	2.2 (1.30–3.80)	0.005∗∗
Treatment failure	Yes	35	9	5.2 (3.39–8.05)	1.9 (1.10–3.30)	0.021∗
	No	52	253	1	1	
Functional status	Appropriate	78	241	1		
	Delayed/Regressing	9	20	1.5 (0.75–3.00)		
Year initiation	<2014	63	157	0.8 (0.51–1.38)		
	≥2014	24	105	1		

∗Significant at <0.05; ∗∗ Significant at <0.01; CHR = Crude hazard ratio; AHR = adjusted hazard ratio; CI = confidence interval, and 1 = reference group.

## 4 Discussion

This study revealed that the median advanced OIs free survival time was 113 months and the overall incidence rate was 5.3 100 person-years among HIV infected children at Debre Tabor referral hospital and University of Gondar Compressive specialized hospital was 5.3 100 person-years.

The incidence of advanced OIs in this study was comparable to the study conducted in the United States of America and meta-analysis in middle and low income countries [31, 32]. However, the incidence of advanced OIs found by this study is lower than the study conducted in Asia (10.5 per 100 person-years) and Ethiopia (9.7 per 100 person-years) [7, 17]. This could be due to the difference in outcome variable criteria. In the above mentioned studies were taking all types of OIs, whereas this study is only advanced OIs. Another explanation may be the differences in follow-up periods, sample size, and study setting.

On the other hand, the incidence of advanced OIs found by this study is higher than the study conducted in Brazil (2.63 per 100 person-years) and Latin America (1.1 per 100 person-years) [18, 33]. The reason for this might be due to the difference in follow-up periods, outcome variable criteria, and study population including adults and children. Moreover, this difference could be due to middle income countries having good healthcare service as compared with resource limited settings like Ethiopia, which can reduce the incidence of advanced OIs.

Pneumonia is the most common (35.6%) during the follow-up time. This finding is consistent with a study conducted in North America, Latin North America, and China [32, 34, 35, 36]. In contrast, a study conducted in Ethiopia and India revealed that TB is a common opportunistic infection [17, 37]. Besides, chronic diarrhea greater than 01 months, which accounts for 10.34% of all types of advanced OIs in this study. This is also a significant finding that hasn't been reported in other studies. Children who were poor/fair level adherence to ART were nearly 2 times more at risk of advanced OIs than those children who were at good level of adherence. This result is consistent with the study conducted in Brazil, Ethiopia, and Cameron [14, 17, 38]. This finding is likely due to the fact that doesn't take ART proper (i.e., poor/fair level of ART) can reduce the higher risk for Advanced OIs and different comorbidity illnesses associated with HIV. Moreover, poor/fair level of ART leading to drug resistance and mortality [38].

Children who were CPT non-user were nearly 2 times more likely to develop advanced OIs as compared to those children with CPT users. This finding is consistent with studies done in Zambia and Ethiopia [9, 17]. This is due to the fact that the main purpose of CPT supplementation is to prevent opportunistic infections associated with HIV.

Children presenting with a low hemoglobin level (10 mg/dl) had a higher risk of developing advanced OIs nearly by three-fold, which is consistent with other studies in Nigeria and Uganda [39, 40]. Besides, children presenting with treatment failure had a higher risk of developing advanced OIs nearly by two-fold. Surprisingly, an IPT non-user and low CD4+ cell count below the threshold level didn't associate with the occurrence of advanced OIs in this study.

In the above mentioned predictor variables (i.e., failure, CPT, non-user, low hemoglobin, and poor/fair level of ART adherence) were less advanced OIs free survival time as compared with their counterparts. The advanced OIs free survival times of children with treatment failure were less free survival time 45 months as compared with hadn't treatment failure history 129 months. Besides, children who were CPT non-user were less advanced OIs free survival time 71 months as compared with CPT users 113 months. Children presenting with good level adherence were higher advanced OIs free survival time 115 as compared with children with poor/fair level ART adherence. Likewise, and children who had hemoglobin level ≥10 mg/dl were higher advanced OIs free survival time 129 as compared with their counterparts 42 months. This is a new significant finding that hasn't been reported in the previous studies era. Hence, prevention and treatment of anemia, treatment failure, and poor/fair level of adherence are pivotal to reduce the incidence of advanced OIs. Alongside, cotrimoxazole preventive therapy supplementation for all children with HIV is the best strategies to reduce the burden of advance OIs.

### 4.1 Limitations of the study

This study is a multicenter with a long (ten-year) follow-up period study. However, some other confounders could affect our results such hygienic practice of the community with awareness level of patients and their caregiver, and easy access to appropriate medical services. Additionally, the data were collected retrospectively or from secondary data, writing bias or bias from interpretation from the medical records may have happened, which might be an underestimated incidence rate of advanced OIs.

## 5 Conclusion

In this study, the median of advanced OIs free survival time was found to be low and the incidence rate was found to be high. The incidence advanced OIs was associated with anemia, treatment failure, and poor/fair level of adherence, cotrimoxazole preventive therapy non-users.

Further research should conduct to evaluate and to improve the quality of care in the study setting.

## Declarations

### Author contribution statement

Ermias Sisay Chanie: Conceived and designed the experiments; Wrote the paper.

Wubet Alebachew Bayih, Binyam Minuye Birhan, Demeke Mesfin Belay, Getnet Asmare, Tegenaw Tiruneh, Yared Asmare Aynalem Aynalem, Biruk Beletew Abat, Sintayehu Asnakew Alemayehu, Maru Mekie, Getache Yideg Yitbarek and Fisha Alebel GebreEyesus: Analyzed and interpreted the data; Contributed reagents, materials, analysis tools or data; Wrote the paper.

### Funding statement

This research did not receive any specific grant from funding agencies in the public, commercial, or not-for-profit sectors.

### Data availability statement

Data included in article/supplementary material/referenced in article.

### Declaration of interests statement

The authors declare no conflict of interest.

### Additional information

No additional information is available for this paper.
